# Temporal and Spatial Expression of Muc1 During Implantation in Sows

**DOI:** 10.3390/ijms11062322

**Published:** 2010-05-27

**Authors:** Qian Ren, Shu Guan, Jinluan Fu, Aiguo Wang

**Affiliations:** College of Animal Science and Technology, China Agricultural University, Beijing 100193, China; E-Mails: qianer0101@gmail.com (Q.R.); guanshu8@gmail.com (S.G.)

**Keywords:** endometrium, expression, implantation, muc1, sows

## Abstract

Recent evidence points to an important role for Muc1 in embryo implantation. In this study, Real-time PCR and immunohistochemistry were used to study mRNA and protein levels at, and between, the attachment sites of the endometrium of Day 13, 18 and 24 pregnant sows. The results indicate that Muc1 mRNA expression was higher between attachment sites than at attachment sites during implantation and this effect was significant on Day 13 (P < 0.01) and 24 (P < 0.01). Intense Muc1 immunostaining was observed in luminal epithelium and stroma and the staining between attachment sites was stronger than at attachment sites on Days 13 and 18. Collectively, these results suggest the crucial role of Muc1 in successful implantation and embryo survival.

## 1. Introduction

Implantation is the process in which mammalian embryos attach to the maternal uterus and interact intimately to form a placenta. The implantation of the porcine embryos into the uterine wall is non-invasive and superficial. It consists of two stages: apposition and adhesion. Porcine embryos begin to attach to the uterus on Day 13 of pregnancy, with attachment complete between Days 18 and 24 [1]. Dantzer found that immobilization of the embryos in the uterus occurs on Days 13–14 of pregnancy and the endometrium formed apical domes on Day 13 in sows [2]. During implantation, the uterus must prepare itself by developing to a receptive state with regard to embryo attachment [3]. Few morphological and molecular correlates of the receptive state are shared among species [4,5]. ‘Markers of receptivity’ which must be displayed by the luminal epithelium, have been applied to identify the receptive state in many species. Uterodome (pinopode) is a morphological marker, which is micro protrusion from the apical uterine epithelium surface [6]. At the molecular level, alteration of protein expression on the cell surface may also contribute to the conversion of the endometrial surface from a non-receptive state to a receptive state [7–10]. Many studies have shown that reduction or loss of Muc1 is a temporal molecular correlate of the receptive state in many species [11–17].

Muc1 is an effective inhibitor of both cell-cell and cell-extracellular matrix (ECM) interactions by steric hindrance [13,15] in both normal and malignant contexts [8,9]. Expression of Muc1 in endometrial epithelium has been suggested to create a barrier to embryo attachment and the barrier must be removed or down-regulated to produce a surface receptive at the time of implantation [18–21].

However, the Muc1 expression and function between/at attachment sites of the porcine uterus throughout the implantation phase were previously not clear. Therefore, the main aim of this study was to detect the expression of Muc1 between, and at, attachment sites of the porcine endometrium in the early, mid- and late stages of embryo implantation. Scanning electron microscopy (SEM) was applied to observe uterodomes in endometrium and to confirm that the sampling sites (between/at attachment sites) were accurate at the beginning of implantantion.

## 2. Results and Discussion

### 2.1. Endometrial Surface Morphological Changes on Day 13 of Pregnancy

**Figure 1.**
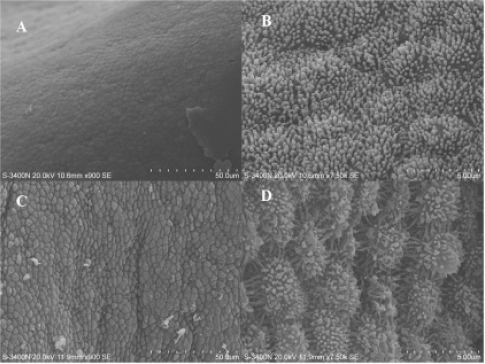
Scanning electron microscope images of the endometrial surface of a Day 13 pregnant sow. (A) and (B) Tissue from between attachment sites. (C) and (D) Tissue at attachment sites.

Endometrial epithelia cells displayed fine microvilli (Figure 1B and 1D) and had a dome-like appearance (Figure 1A and 1C). Between attachment sites, uterodomes were very small, or completely absent. At attachment sites, uterodomes were well formed and isolated. Microvilli membrane were developed and expanded. Uterodomes are morphological markers for endometrial receptivity which indicate the opening of the “implantation window” [22]. Porcine embryos begin to attach to the uterus on Days 13–14 of pregnancy [1,2]. SEM was used to examine morphology of endometrial surfaces on Day 13 of pregnancy in sows. We can be sure that Day 13 of pregnancy is the early stage of implantation in the selected sows since uterodome formations were observed. Uterodomes are micro protrusions from the apical uterine epithelium surface, which inter-digitate with microvilli on the apical syncytiotrophoblast surface of the blastocyst. We found the formation and development of uterodomes at attachment sites occur before between attachment sites. These observations suggested that the sampling sites (between/at attachment sites) were accurate though the embryo could not be observed on day 13 of pregnancy.

### 2.2. Tissue Distribution of Muc-1 mRNA in a Sow

**Figure 2.**
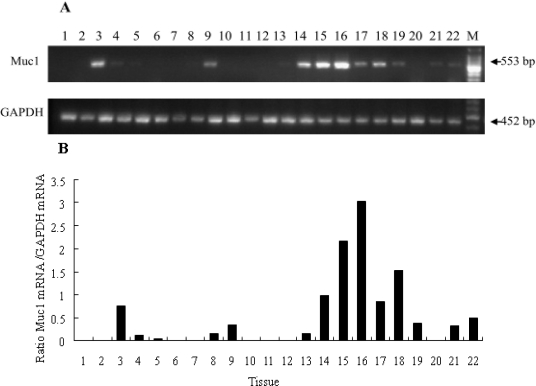
The Semi-Quantitative Polymerase Chain Reaction (SQ-PCR) analysis of Muc1 mRNA in porcine tissues (Day 24 of pregnancy). (A) mRNA products of SQ-PCR analysis. A single amplified fragment of 553 bp was detected for Muc1 and 452 bp for GAPDH. GAPDH was amplified to quantify and test quality of cDNA. M: 100 bp molecular Marker. Expected fragment length (bp) is indicated on the right. (B) Relative abundance of Muc1 mRNA normalized to GAPDH. 1: heart; 2: spleen; 3: kidney; 4: spinal cord; 5: bladder; 6: adrenal gland; 7: brain; 8: cerebellum; 9: pituitary; 10: hypothalamus; 11: back subcutaneous fat; 12: skeletal muscle; 13: ovary; 14: oviduct; 15: body of uterus; 16: cervix; 17: endometrium (at attachment sites); 18: endometrium (between attachment sites); 19: embryo; 20: liver; 21: lung; 22: large intestine.

Relative abundance of Muc1 mRNA was assessed in various tissues from a pregnant sow (Day 24 of pregnancy). The expression of Muc1 differed significantly among tissues. No expression was observed in heart, spleen, adrenal gland, brain, hypothalamus, liver, skeletal muscle or back subcutaneous fat. The highest expression was obtained in the cervix tissue (Figure 2B). The expression of Muc1 mRNA was highly enriched in the female reproductive tract and embryo, suggesting a putative role for Muc1 in reproductive tissues. Previous studies reported that the MUC1 (human) protein was expressed in the female reproductive tract [23]. However, no Muc1 mRNA expression was detected in porcine heart, spleen, adrenal gland, brain, hypothalamus, liver, skeletal muscle or back subcutaneous fat, which was in accordance with the mouse [24]. The phenomenon that Muc1 mRNA expression varies significantly among tissues implies that Muc1 functions in different tissues.

### 2.3. Differential Expression of Muc1 in Porcine Endometrium

**Figure 3.**
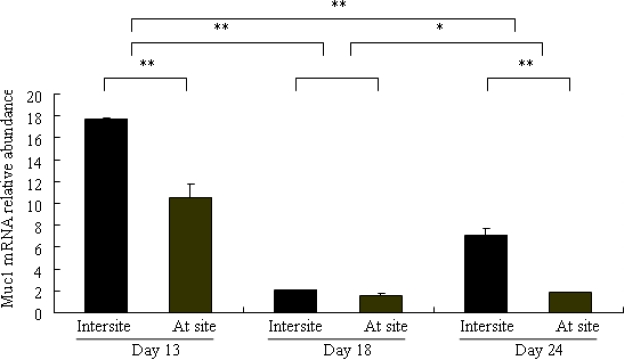
Effects of the day of pregnancy and site of endometrial tissue sampling on the relative expression of Muc1 mRNA in endometrial tissue. Data are ratios of Muc1 relative mRNA abundance normalized to GAPDH. At site: endometrial tissue sample taken at attachment sites; Intersite: endometrial tissue sample taken between attachment sites. Each bar represents means ± SEM; *P < 0.05, * *P < 0.01.

The effect of the day of pregnancy on Muc1 mRNA expression in the porcine endometrium during the embryo implantation is shown in Figure 3. The expression in pregnant sows was highest by Day 13, as compared with Day 18 (P < 0.01) and 24 (P < 0.01). There were significant differences between Days 18 and 24 pregnant sows (P < 0.05). To determine whether porcine Muc1 mRNA expression could be modulated according to the site of endometrial tissue sampling, tissues were collected at and between attachment sites. Expression was higher between attachment sites compared with at attachment sites, and this effect was significant at Day 13 (P < 0.01) and 24 (P < 0.01) of pregnancy. In contrast, there was no effect of endometrial tissue site sampling at Day 18 of pregnancy (P > 0.05).

**Figure 4.**
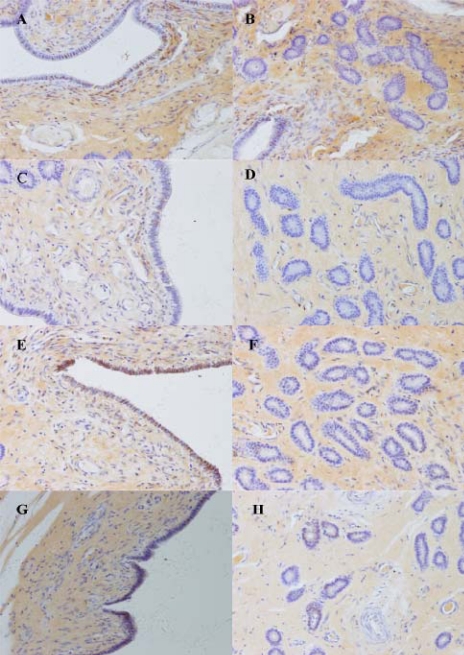

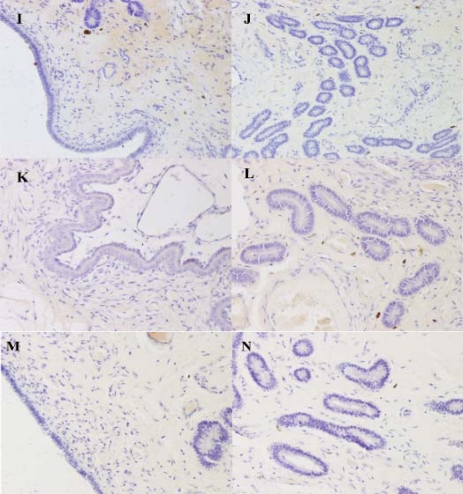
Immunohistochemical localization of Muc1 in pig uterus. (A) and (B): Tissue from between attachment sites of a Day 13 pregnant sow. (C) and (D): At attachment sites of a Day 13 pregnant sow. (E) and (F): Between attachment sites of a Day 18 pregnant sow. (G) and (H): At attachment sites of a Day 18 pregnant sow. (I) and (J): Between attachment sites of a Day 24 pregnant sow. (K) and (L): At attachment sites of a Day 24 pregnant sow. (M) and (N): At attachment sites of a Day 13 pregnant sow, negative controls for localization (×200).

The expression of Muc1 protein between and at attachment sites on Days 13, 18 and 24 of pregnancy are summarized in Table 1. On Day 13 of pregnancy, Muc1 staining between attachment sites was strong in the luminal epithelium and subepithelial stroma, but weak in the glandular epithelium (Figure 4A and 4B). At attachment sites, staining was moderate in the luminal epithelium and stroma, but absent in the glandular epithelium (Figure 4C and D). On Day 18 of pregnancy, very strong staining was observed in the luminal epithelium, and moderate staining in the glandular epithelium and stroma were detected between attachment sites (Figure 4E and F). At the attachment sites, staining was strong in the luminal and glandular epithelium, but weak in the stroma (Figure 4G and H). On Day 24 of pregnancy, staining was absent in the luminal epithelium, glandular epithelium and stroma (Figure 4I–L). A minimal background, but no staining, was seen in the negative controls (Figure 4M and N).

In this study, Muc1 was detected both at transcript and protein level in porcine endometrium. Muc1 abundance varied with the day of pregnancy and the site of endometrial tissue sampling. The above evidence suggests the important role of this gene in implantation of sows. The expression of Muc1 mRNA and protein in porcine endometrium between attachment sites was higher than at attachment sites during implantation. A similar expression pattern was also observed on Day 7.25 postcoitum in rabbits [25]. These findings demonstrate that reduction of Muc1 expression at attachment sites may cause enhancement of endometrial receptivity, and result in successful implantation of embryos. Furthermore, local loss of Muc1 may involve both a stimulation of Muc1 protein turnover and a reduction in *de novo* synthesis. Bowen *et al*. reported that Muc1 was not detected in porcine luminal epithelium at attachment sites on Days 10–15 of pregnancy [26]. In contrast with this result, we found moderate staining on Days 13. The difference may be due to differences in the cytochemical approaches, antibodies, and experimentation design. At attachment sites, Muc1 mRNA expression in endometrium was highest on Day 13 and it decreased on Days 18 and 24. Muc1 protein was mainly localized in the luminal epithelia and the staining was strong on Day 18, moderate on Day 13, but absent on Day 24. Since biopsy samples in this research included glandular, luminal epithelia, storma and myometrium, the Muc1 mRNA expression could not compare with protein expression in different regions.

In *in vitro* models of human, MUC1 was present in primary cultures of human endometrial epithelial cells (EEC). Presence of a human blastocyst (*i.e.*, apposition phase) increased EEC MUC1 protein, compared with control EEC lacking embryos. When human blastocysts were allowed to attach to the EEC monolayer (*i.e.,* adhesion phase), MUC1 was locally removed in a paracrine fashion on EEC at attachment sites [27]. In *in vitro* models of rabbits, luminal epithelium apposed to blastocysts had a marked reduction or absence of Muc1 immunostaining [26]. In our study, Muc1 protein expression in the luminal epithelium increased in mid-implantation (Day 18) and was removed in late implantation (Day 24) in pig. The results imply that the presence of blastocysts results in a localized down-regulation of Muc1 expression, and the loss of Muc1 in porcine luminal epithelium at attachment sites on Day 24 suggests that the uterus has greater receptivity in late implantation. The mechanism of this type of regulation remains to be established.

In this study, immunostaining for Muc1 in the endometrial stroma underlying the luminal epithelium was observed during early and mid-implantation, and higher level expression between attachment sites compared with at attachment sites. In mice, Muc1 immunopositive reaction was found in the deciduas by Day 8 of pregnancy onwards [18]. The observed pattern was unusual, because Muc1 is considered to be an epithelial differentiation marker, and this is the first report of its expression by non-epithelial cells. Porcine embryos undergo true epitheliochorial placentation in which the luminal epithelium remains morphologically intact and the embryos trophectoderm simply attaches to the apical luminal epithelium surface without displacement or invasion of uterine stromal cells [28]. Lin *et al*. and Johnson *et al*. reported a stromal decidualization-like response in the pregnant ovine and porcine uterus by studying osteopontin, integrin αV and β3 expression [29,30]. In our study, a similar phenomenon was found. Porcine embryos do not invade the uterine wall. However, Muc1 is expressed in stroma and it reduces at attachment sites compared with between attachment sites during early, and mid-stages of implantation. This phenomenon indicates the important role of Muc1 in conceptus survival, since stroma is crucial for maintaining morphogenesis, hormonal responsiveness, and secretory function of the uterine epithelium [31,32]. Moreover, epithelial-stromal interactions have been implicated in development, growth, differentiation, and adult function of the uterus [33]. So there may be a decidualization-like response in pregnant porcine uterus stroma, though the degree is lower.

On Day 13, appearance of uterodomes signaled the opening of the window of implantation. We found reduction of Muc1 mRNA expression and protein expression in the luminal epithelium accompanied with well-formed uterodomes at attachment sites. In contrast, there was higher expression of Muc1 with small or absent uterodomes between attachment sites. The reason can be explained by that developing uteridomes consistent with reduction of Muc1 expression in endometrium may be ready to implant at this stage, and the variation can indicate the receptivity of uterus at the window of implantation.

## 3. Experimental Section

### 3.1. Animals and Tissue Collection

#### 3.1.1. Animals

Multiparous Yorkshire sows (5th parity) were observed daily for estrous behavior in the presence of a boar. Sows exhibiting at least two estrous cycles of normal duration (21 days) were inseminated twice, 12 and 24 h after estrus detection. Fifteen sows were slaughtered (n = 5/day) at different stages of early pregnancy by electrical stunning on Days 13, 18 and 24 of pregnancy. The day of 24 h after estrus detection was considered Day 0. The slaughter was conducted according to procedure of Animal Welfare Committee in China Agricultural University.

#### 3.1.2. Tissue Collection

Endometrial tissue samples were prepared according to the procedure of Lord with minor modifications [34]. Several sections of each uterine horn of sows from each state were collected immediately. For SEM, specimens (2 mm^3^) were fixed in 5 % (v/v) glutaraldehyde in 0.1 M phosphate buffer (pH 7.4) for 4 h at room temperature. For immunohistochemistry, specimens (1.5 cm^3^) were fixed in 4 % (w/v) paraformaldehyde in PBS (pH 7.4) at 4 °C overnight, paraffin embedded, sectioned, and stained with haematoxylin-eosin. For RNA extraction, specimens were placed in RNAlater (Qiagen, Valencia, CA, USA) at 4 °C overnight and then stored at −20 °C.

Several tissues of a sow (Day 24 of pregnancy) were sampled for RNA extraction, including heart, spleen, kidney, spinal cord, bladder, adrenal gland, brain, cerebellum, pituitary, hypothalamus, back subcutaneous fat, skeletal muscle, ovary, oviduct, body of uterus, cervix, embryo, liver, lung and large intestine.

### 3.2. Scanning Electron Microscopy (SEM)

Scanning Electron Microscopy (SEM) was performed on the exposed maternal endometrial surfaces of sows (Day 13 of pregnancy). Tissues were processed using the method of Abd-Elnaeim [35], with minor modifications. Small pieces of tissue were fixed in 5% (v/v) glutaraldehyde in 0.1 M phosphate buffer (pH 7.4) for 4 h at room temperature. Then, the tissues were post-fixed in 2 % OsO_4_ (w/v) in 0.1 M phosphate buffer (pH 7.4) for 1 h at room temperature. After dehydration by a series of aqueous solutions of ethanol (50 to 100 % v/v), isoamylacetate substitution was done. The specimens were critical point dried using CO_2_-substitution, mounted on aluminium stubs, sputter-coated with gold, and examined and photographed using a Hitachi S-3400N (Hitachi, Tokyo, Japan) scanning electron microscope.

### 3.3. RNA Extraction and Reverse Transcription

Trizol reagent (InVitrogen, Carlsbad, CA, USA) was used to extract total RNA according to the manufacturer’s instructions, and was kept at −80 °C until used. The purity and integrity of RNA was electrophoretically tested by ethidium bromide staining, optical density (OD) absorption ratio OD_260_/OD_280_ (>1.90) and rRNA (28s/18s) ratios (≈2), respectively. Two micrograms of total RNA were reverse-transcribed into cDNA in the presence of polythymidine oligonucleotide primers (Oligo-dT18) and Moloney Murine Leukemia Virus Reverse Transcriptase (M-MLVRT; Promega, Madison, WI, USA) in a total reaction volume of 25 μl. RT products were stored at −20 °C for use.

### 3.4. Primer Design

The mRNA sequence of porcine epithelial mucin (Muc1; GenBank AY243508) was used to design two pairs of primers (Table 2). To ensure amplification of only the complementary DNA (cDNA) and not the genomic DNA (gDNA), the forward and reverse primers used for amplification were placed in two different exons of the gene, and they were all directly against the highly conserved region of the sequence. Primers were designed using Primer express software v. 3.0 (Applied Biosystems, Foster City, CA, USA). Housekeeping gene glyceraldehyde 3-phosphate dehydrogenase (GAPDH) was assayed as normalization control to correct for loading discrepancies for all samples assayed. Primer for GAPDH-1 was provided by BDBiosciences (Bedforld, USA); Primer for GAPDH-2 was used according to Lin *et al.* [29]. They are listed in Table 2. Primers were synthesized by Shanghai Sangon Biological Engineering Technology And Service Co., Ltd (Beijing, China).

### 3.5. Semi-Quantitative Polymerase Chain Reaction (SQ-PCR)

The presence of mRNAs for Muc1 in several tissues of a pregnant sow (Day 24 of pregnancy) was examined by SQ-PCR. Repeated experiments were carried out to determine the optimal cycle number for each gene to ensure the analyses were performed at the exponential phase of amplification, before the saturation level was reached. PCR was carried out in 25 μl reaction volumes. Each reaction contained: 2 μl cDNA template, 2.5 μl 10×PCR buffer (containing 100 mM Tris-HCl (pH 8.0), 500 mM KCl, 10 mM of MgCl_2_ and 0.1 % glutin), 2.0 μl 2.5 mM dNTPs Mix, 0.5 μl forward and reverse primers each (10 pmol/μl), 0.5 μl AmpliTaq DNA polymerase (5 U/μl), and 17 μl double distilled water (The reagents all came from State Key Laboratory for Agri-biotechnology, China Agricultural University, China). The amplifying conditions of PCR were 94 °C for 5 min, followed by 30 cycles (27 cycles for GAPDH) of 94 °C for 30 sec, annealing for 30 sec, 72 °C for 30 sec, and then 72 °C for 7 min. The annealing temperatures for Muc1 and GAPDH were 62 °C and 60 °C, respectively. Equal amounts of PCR products were loaded per lane and electrophoresed on a 1.2% agarose gel, stained with ethidium bromide. The gel image was scanned and recorded using a Gel Doc XR (Bio-Rad, Hercules, CA, USA) imaging system. The identity of each amplified PCR product was verified by sequence analysis. The intensity of the bands was quantified by densitometry analysis using “Quantity One” software (Bio-Rad, Hercules, CA, USA). Relative abundance of Muc1 mRNA was normalized by GAPDH.

### 3.6. Real-Time Polymerase Chain Reaction (RT-PCR)

RT-PCR was performed with an ABI Prism 7900HT (Applied Biosystems, Foster City, CA, USA) Sequence Detection System using SYBR green PCR master mix (Applied Biosystems) to analyze Muc1 expression in endometrium of sows (Days 13, 18 and 24 of pregnancy). Reactions were prepared in 25 μl volume consisting of 1.5 μl RT product, 0.5 μl forward and reverse primers each (10 pmol/μl), 12.5 μl SYBR green PCR master mix and 10 μl double distilled water. PCR thermal cycling conditions were 50 °C for 2 min, and 95 °C for 10 min, followed by 40 cycles of 95 °C for 15 sec and 60 °C for 30 sec. After each PCR reaction, melting curves were obtained by stepwise increases in the temperature from 60 to 95 °C to ensure single product amplification. In PCR reactions, RNA and gDNA were used as negative and positive controls, respectively, and no amplicons were obtained by using RNA directly. All samples were measured in triplicates. The identity of PCR products were verified by sequence analysis after cloning into the pMD 18-T vector (TaKaRa, Dalian, China). Relative abundance of Muc1 mRNA normalized to GAPDH was analyzed by 2^−^^ΔCt^ comparative C_t_ method [36,37].

### 3.7. Immunohistochemistry

Immunohistochemistry was performed by the labeled streptavidin/peroxidase biotin method (Zymed, South San Francisco, CA, USA) to analyze Muc1 expression in endometrium of sows (Days 13, 18, and 24 of pregnancy). The tissue sections were cut at 4 μm thickness and mounted on silanized slides, dewaxed in xylene, and rehydrated in graded ethanol. Sections were treated with 3 % H_2_O_2_ in PBS for 10 min to quench endogenous peroxidase activity. Sections were then incubated in 5 % goat serum in PBS for 20 min to reduce nonspecific binding. After tapping the excess goat serum solution, sections were incubated overnight at 4 °C with a mouse monoclonal antibody against full length human MUC1 (sc-59794, Santa Cruz, California, CA, USA; Immunoblotting of porcine uterus extractions using this primary antibody was performed to confirm the specificity of this antibody in pig) diluted 1:200, then incubated for 20 min in biotinylated goat anti-mouse antibody (Zymed), followed by incubation with HRP-streptavidin (Zymed) for 20 min. The antibody binding sites were visualized by incubating the tissue sections with DAB solution provided by a DAB kit (Zymed). Finally, sections were counterstained with haematoxylin, dehydrated, and mounted. For the negative controls mouse antibody (Zymed) was used at the same concentration as primary antibodies. Images of the sections were captured using Olympus microscope BX51 and digital camera DP70 (Olympus, Tokyo, Japan) and were quantified visually as absent (−), weak (±), moderate (+), strong (++) or very strong (+++) according to the intensity and density of stained cells.

### 3.8. Statistical Analysis

The data were expressed as means ± SEM. The statistical comparison of relative mRNA expression of Muc1 between experimental groups were analyzed by all pair-wise multiple comparison procedures (Tukey test) or by a two-way ANOVA (full factorial on sampling site and day of pregnancy) where pertinent. Data were analyzed using Statistical Analysis Software Version 8.02 (SAS; SAS Institute, Cary, NC, USA). Statistical significance was determined at p < 0.05.

## 4. Conclusions

Muc1 plays an important role during successful embryo implantation. To explore the implantation mechanism, we can focus on the function of Muc1 in endometrial stroma in pig, since pig is the only species that demonstrates a true epitheliochorial placental animal.

## Figures and Tables

**Table 1.** Expression of Muc1 protein in porcine endometrium on Days 13, 18 and 24 of pregnancy.

	**Day 13**			**Day 18**			**Day 24**		
	
	**LE**	**GE**	**S**	**LE**	**GE**	**S**	**LE**	**GE**	**S**
intersite	++	±	++	+++	+	+	−	−	−
At site	+	−	+	++	++	±	−	−	−

Note: − absent; ± weak; + moderate; ++ strong; +++ very strong.

LE = luminal epithelium; GE = glandular epithelium; S = stroma.

At site = at attachment site; Intersite = between attachment sites.

**Table 2.** Oligonucleotide primers used for semi-quantitative PCR (SQ-PCR) and real-time PCR (RT-PCR) of porcine Muc1 and a house keeping gene.

**Primer Name**	**Primer Sequences (5′–3′)**	**Annealing Temperature (**°C**)**	**Product Size (bp)**	**Genbank Accession no./references**
SQ-PCR				
Muc1-1	Forward:CACCACCAGCTACTACAAGG	62	553	AY243508
	Reverse:TGCCAGGTTCGAGTAAGAG			
GAPDH-1	Forward:ACCACAGTCCATGCCATCAC	60	452	AF017079/BDBiosciences
	Reverse:TCCACCACCCTGTTGCTGTA			
RT-PCR				
Muc1-2	Forward:GTGCCGACGAAAGAACTG	60	187	AY243508
	Reverse:TGCCAGGTTCGAGTAAGAG			
GAPDH-2	Forward:GTCCACTGGTGTCTTCACGA	60	154	AF141959/[29]
	Reverse:GCTGACGATCTTGAGGGAGT			

## References

[1] Kyriazakis I Whittemore CT Whittemore’s Science and Practice of Pig Production3rd ed Blackwell Publishing Oxford, UK 2006 105 147

[2] Dantzer V Electron microscopy of the initial stages of placentation in the pig Anat. Embryol 1985 172 281 293 4061869 10.1007/BF00318976

[3] Psychoyos A Uterine receptivity for nidation Ann. N.Y. Acad. Sci 1986 476 36 42 3541745 10.1111/j.1749-6632.1986.tb20920.x

[4] Carson DD Bagchi I Dey SK Enders AC Fazleabas AT Lessey BA Yoshinaga K Embryo implantation Dev. Biol 2000 223 217 237 10882512 10.1006/dbio.2000.9767

[5] Sharkey AM Smith SK The endometrium as a cause of implantation failure Best Pract. Res. Clin. Obstet. Gynaecol 2003 17 289 307 12758101 10.1016/s1521-6934(02)00130-x

[6] Psychoyos A Nikas G Uterine pinopodes as markers of uterine receptivity Assist. Reprod. Rev 1994 4 26 32

[7] Acosta AA Elberger L Borghi M Calamera JC Chemes H Doncel GF Kliman H Lema B Lustig L Papier S Endometrial dating and determination of the window of implantation in healthy fertile women Fertil. Steril 2000 73 788 798 10731542 10.1016/s0015-0282(99)00605-6

[8] Aplin JD MUC-1 glycosylation in endometrium: possible roles of the apical glycocalyx at implantation Hum. Reprod 1999 14 Suppl 2 17 25 10690797 10.1093/humrep/14.suppl_2.17

[9] Giudice LC Potential biochemical markers of uterine receptivity Hum. Reprod 1999 14 Suppl 2 3 16 10690796 10.1093/humrep/14.suppl_2.3

[10] Horne AW White JO Lalani EN The endometrium and embryo implantation. A receptive endometrium depends on more than hormonal influences BRIT. MED. J 2000 321 1301 1302 11090499 10.1136/bmj.321.7272.1301 PMC1119051

[11] Carson DD DeSouza MM Kardon R Zhou X Lagow E Julian J Mucin expression and function in the female reproductive tract Hum. Reprod. Update 1998 4 459 464 10027596 10.1093/humupd/4.5.459

[12] Carson DD DeSouza MM Regisford EG Mucin and proteoglycan functions in embryo implantation Bioessays 1998 20 577 583 9723007 10.1002/(SICI)1521-1878(199807)20:7<577::AID-BIES9>3.0.CO;2-H

[13] Gendler SJ Spicer AP Epithelial mucin genes Annu. Rev. Physiol 1995 57 607 634 7778880 10.1146/annurev.ph.57.030195.003135

[14] Hanisch FG Muller S MUC1: The polymorphic appearance of a human mucin Glycobiology 2000 10 439 449 10764832 10.1093/glycob/10.5.439

[15] Lagow E DeSouza MM Carson DD Mammalian reproductive tract mucins Hum. Reprod. Update 1999 5 280 292 10465520 10.1093/humupd/5.4.280

[16] Simon C Valbuena D Embryonic implantation Ann. Endocrinol. (Paris) 1999 60 134 136 10456186

[17] Thathiah A Carson DD Mucins and blastocyst attachment Rev. Endocr. Metab. Disord 2002 3 87 96 12007285 10.1023/a:1015446626671

[18] Braga VM Gendler SJ Modulation of Muc-1 mucin expression in the mouse uterus during the estrus cycle, early pregnancy and placentation J. Cell. Sci 1993 105 Pt 2 397 405 7691839 10.1242/jcs.105.2.397

[19] Chervenak JL Illsley NP Episialin acts as an antiadhesive factor in an *in vitro* model of human endometrial-blastocyst attachment Biol. Reprod 2000 63 294 300 10859271 10.1095/biolreprod63.1.294

[20] DeSouza MM Surveyor GA Price RE Julian J Kardon R Zhou X Gendler S Hilkens J Carson DD MUC1/episialin: A critical barrier in the female reproductive tract J. Reprod. Immunol 1999 45 127 158 10674981 10.1016/s0165-0378(99)00046-7

[21] Surveyor GA Gendler SJ Pemberton L Das SK Chakraborty I Julian J Pimental RA Wegner CC Dey SK Carson DD Expression and steroid hormonal control of Muc-1 in the mouse uterus Endocrinology 1995 136 3639 3647 7628404 10.1210/endo.136.8.7628404

[22] Martel D Malet C Gautray J Psychoyos A The Endometrium, Hormonal Impacts Plenum Press New York, NY, USA 1981 15

[23] Brayman M Thathiah A Carson DD MUC1: A multifunctional cell surface component of reproductive tissue epithelia Reprod. Biol. Endocrinol 2004 2 4 14711375 10.1186/1477-7827-2-4 PMC320498

[24] Pemberton L Taylor-Papadimitriou J Gendler SJ Antibodies to the cytoplasmic domain of the MUC1 mucin show conservation throughout mammals Biochem. Biophys. Res. Commun 1992 185 167 175 1599454 10.1016/s0006-291x(05)80971-4

[25] Hoffman LH Olson GE Carson DD Chilton BS Progesterone and implanting blastocysts regulate Muc1 expression in rabbit uterine epithelium Endocrinology 1998 139 266 271 9421424 10.1210/endo.139.1.5750

[26] Bowen JA Bazer FW Burghardt RC Spatial and temporal analyses of integrin and Muc-1 expression in porcine uterine epithelium and trophectoderm *in vivo* Biol. Reprod 1996 55 1098 1106 8902223 10.1095/biolreprod55.5.1098

[27] Meseguer M Aplin JD Caballero-Campo P O’Connor JE Martin JC Remohi J Pellicer A Simon C Human endometrial mucin MUC1 is up-regulated by progesterone and down-regulated *in vitro* by the human blastocyst Biol. Reprod 2001 64 590 601 11159362 10.1095/biolreprod64.2.590

[28] Burghardt RC Johnson GA Jaeger LA Ka H Garlow JE Spencer TE Bazer FW Integrins and extracellular matrix proteins at the maternal-fetal interface in domestic animals Cells Tissues Organs 2002 172 202 217 12476049 10.1159/000066969

[29] Lin HC Wang X Liu GF Fu JL Wang AG Expression of alphaV and beta3 integrin subunits during implantation in pig Mol. Reprod. Dev 2007 74 1379 1385 17440962 10.1002/mrd.20732

[30] Johnson GA Burghardt RC Joyce MM Spencer TE Bazer FW Pfarrer C Gray CA Osteopontin expression in uterine stroma indicates a decidualization-like differentiation during ovine pregnancy Biol. Reprod 2003 68 1951 1958 12606396 10.1095/biolreprod.102.012948

[31] Cunha GR Bigsby RM Cooke PS Sugimura Y Stromal-epithelial interactions in adult organs Cell Differ 1985 17 137 148 3902250 10.1016/0045-6039(85)90481-6

[32] Cunha GR Chung LW Shannon JM Taguchi O Fujii H Hormone-induced morphogenesis and growth: role of mesenchymal-epithelial interactions Recent Prog. Horm. Res 1983 39 559 598 6314450 10.1016/b978-0-12-571139-5.50018-5

[33] Guillomot M Flechon JE Wintenberger-Torres S Conceptus attachment in the ewe: An ultrastructural study Placenta 1981 2 169 182 7232339 10.1016/s0143-4004(81)80021-5

[34] Lord E Murphy BD Desmarais JA Ledoux S Beaudry D Palin MF Modulation of peroxisome proliferator-activated receptor delta and gamma transcripts in swine endometrial tissue during early gestation Reproduction 2006 131 929 942 16672357 10.1530/rep.1.00657

[35] Abd-Elnaeim MM Saber A Hassan A Abou-Elmagd A Klisch K Jones CJ Leiser R Development of the areola in the early placenta of the one-humped camel (Camelus dromedarius): A light, scanning and transmission electron microscopical study Anat. Histol. Embryol 2003 32 326 334 14651479 10.1111/j.1439-0264.2003.00465.x

[36] Livak KJ Schmittgen TD Analysis of relative gene expression data using real-time quantitative PCR and the 2(-Delta Delta C(T)) Method Methods 2001 25 402 408 11846609 10.1006/meth.2001.1262

[37] Schmittgen TD Livak KJ Analyzing real-time PCR data by the comparative C(T) method Nat. Protoc 2008 3 1101 1108 18546601 10.1038/nprot.2008.73

